# The Profiles of Long Non-coding RNA and mRNA Transcriptome Reveals the Genes and Pathway Potentially Involved in *Pasteurella multocida* Infection of New Zealand Rabbits

**DOI:** 10.3389/fvets.2021.591273

**Published:** 2021-05-05

**Authors:** Jiaqing Hu, Wenqiang Li, Bing Huang, Qiaoya Zhao, Xinzhong Fan

**Affiliations:** ^1^Shandong Provincial Key Laboratory of Animal Biotechnology and Disease Control and Prevention, College of Animal Science and Veterinary Medicine, Shandong Agricultural University, Taian, China; ^2^Shandong Provincial Key Laboratory of Poultry Disease Diagnose and Immune, Institute of Poultry, Shandong Academy of Agricultural Sciences, Jinan, China

**Keywords:** long non-coding RNA, gene expression, *Pasteurella multocida* attacks, high throughput sequencing, functional enrichment, immune response

## Abstract

Infection with *Pasteurella multocida* (*P. multocida*) causes severe epidemic diseases in rabbits and is responsible for the pronounced economic losses in the livestock industry. Long non-coding RNAs (lncRNAs) have been proven to exert vital functions in regulating the host immune responses to bacterial attacks. However, little is known about how lncRNAs participate in the rabbit's immune response against *P. multocida* infection in the lungs. LncRNA and mRNA expression profiles were analyzed by transcriptomics and bioinformatics during *P. multocida* infection. A total of 336 lncRNAs and 7,014 mRNAs were differentially regulated at 1 day and 3 days post infection (dpi). Nearly 80% of the differentially expressed lncRNAs exhibited an increased expression at 3 dpi suggesting that the *P. multocida* genes are responsible for regulation. Moreover, GO and KEGG enriched analysis indicated that the immune-related pathways including pattern recognition receptors (PRRs), cytokines, and chemokines were significantly enriched at 3 dpi. These results indicate that the dysregulated immune-related genes may play crucial roles in defending against *P. multocida* attacks. Overall, these results advance our cognition of the role of lncRNAs and mRNAs in modulating the rabbit's innate immune response against *P. multocida* attacks, which will offer a valuable clue for further studies into exploring *P. multocida*-related diseases in human.

## Introduction

*Pasteurella multocida* (*P. multocida*) is a notorious Gram-negative opportunistic pathogen that is ubiquitous in the respiratory tracts of different animal species and results in enormous economic losses (1, 2). It can be sorted into five serogroups (A, B, D, E, and F) and 16 Heddleston serotypes based on its capsular antigens and lipopolysaccharide (LPS) antigens (2, 3). In rabbits, *P. multocida* is a predominant cause of death with a spectrum of hemorrhagic septicemia and respiratory problems (1, 4). Notably, the prevalence of *P.multocida* has been reported as 94%, and even most adult rabbits are considered to be infected with *P. multocida* (5). Human cases of *P. multocida* infections, which have been transmitted via licking or biting from rabbits, have raised substantial concern (6, 7). However, the molecular pathogenesis of the disease remains unclear.

With advances in the next-generation RNA sequencing (RNA-seq), it is possible to quantify both coding and non-coding RNAs (ncRNAs) across a considerably dynamic range, and it is a forceful tool to accurately identify the differential expression (DE) of mRNAs and ncRNAs (8). Advances in RNA-seq have also revealed that < 2% of the mammalian genome is transcribed into protein-coding mRNAs, while the remaining genome produces numerous ncRNAs (9). ncRNAs were deemed as junk molecules of transcription, and it has recently been discovered and proven that they exert an essential function in diverse cellular physiological and pathological processes. Based on their sequence-length cutoff of 200 nucleotides, ncRNAs are subdivided into small ncRNAs, such as microRNAs (miRNAs) and long ncRNAs (lncRNAs). These ncRNAs regulate the gene expression at the levels of pre-transcription, transcription, and post-transcription (10). To date, RNA-seq technology combined with bioinformatics analysis have yielded countless DE mRNAs and ncRNAs, which act important roles in understanding the complex molecular mechanism of host-pathogen interactions (11, 12).

LncRNAs are defined as transcribed RNA molecules that are more than 200 nucleotides long and lack protein-coding potential. Generally, they can be divided into five distinct subtypes based on the position and direction to nearby protein-coding genes: sense, antisense, intergenic, intronic, and bidirectional (13). LncRNAs are potent regulators that directly interact with other RNAs, proteins, or chromatin to govern gene expression and function through distinct mechanisms, including signaling, scaffolds, decoys, and competitive endogenous RNAs (CeRNA) (14, 15). Accumulating shreds of evidence have clarified that these lncRNAs combined with mRNAs contribute to numerous biological processes, especially in the immune response of host-pathogen interactions against various infectious agents (12). For example, the long intergenic ncRNA (lincRNA)-cyclooxygenase 2 (Cox2) was found to trans-regulate both the activation and repression of distinct classes of immune response genes in bone marrow-derived dendritic cells upon toll-like receptor (TLR) ligand stimuli (16). The lincRNA THRIL (TNFα and hnRNPL related immunoregulatory LincRNA) is capable of modulating the expression of the proinflammatory cytokine TNF-α in trans by forming a complex with the hnRNPL (heterogeneous nuclear ribonucleoprotein L) that functions at the TNF-α promoter upon TLR1/2-stimulated THP1-derived human macrophages (17). Meanwhile, multiple TLR ligands induced the lnc MARCKS (myristoylated alanine-rich protein kinase C) production to the master regulator of inflammatory responses interacted with APEX1 (apurinic/apyrimidinic endodeoxyribonuclease 1) to form a hnRNPL complex at the MARCKS promoter (18). Recently, an increasing number of lncRNAs have been identified, and their functions in the host-pathogen interactions of humans, mice, and pigs have been clarified (12). However, the alteration in the lncRNA expression induced by rabbit *P. multocida* remains unclear, as do the roles of these transcripts in modulating the lung response to infection remain unclear.

In our research, we depicted the lncRNA and mRNA profiles of rabbit lungs infected with *P. multocida* for 1 day (P1) and 3 days (P3) or without *P. multocida* (control, P0). This study aimed to obtain the DE transcripts (lncRNAs and mRNAs) during the *P. multocida* insults with the aim of exploring the primary biological processes and pathways associated with the pathogenesis mechanisms. Thus, the data obtained from this research could be used to establish different cellular and molecular events upon the *P. multocida* attack, which could contribute to uncovering the molecular pathogenesis implying the *P. multocida* attack. The findings of this study also provide a valuable reference for further study on lncRNAs concerning *P. multocida*-related diseases in human.

## Materials and Methods

### *Pasteurella multocida* Isolation, Identification, Pathogenicity, and Infection

The strain was isolated from a suspected rabbit lung infected with *P. multocida* and stored in our lab. Then, the recovered strain was inoculated onto 5% defibrinated sheep blood agar and MacConkey agar and incubated at 37°C for 24 h. A presumptive *P. multocida* colony was preliminarily identified using routine biochemical testing and API 20 E strips (bioMérieux, Durham, NC, USA), and further confirmed by PCR assay to amplify the inherent gene according to the published articles (19).

All animal studies in this article were performed according to the guidelines of the Institutional Animal Care and Use Committee of Shandong Agricultural University and the “Guidelines for Experimental Animals” of the Ministry of Science and Technology (Beijing, China).

New Zealand rabbits that were 38-days-old were purchased from Qingdao Kangda Rabbit Industry Development Co., Ltd. They were tested negative for *P. multocida* by separating them from the nares. The detected samples were collected by rotating a sterile cotton swab three times in both anterior nares. We also tested negative for *P. multocida* by detecting the antibodies against *P. multocida* in their serum applying a commercial ELISA assay. Blood samples were obtained from the marginal vein of the rabbit ear and serum was separated from the blood using a centrifuge (1,000 rpm, 10 min). All the rabbits were adapted to the experimental environment for seven days, and then the experiment was conducted.

The bacteria were quantified using a plate counting method and diluted in PBS at a final concentration of 1 × 10^9^ colony forming units (CFU)/mL. Eight 45-days-old New Zealand rabbits that were confirmed free of *P. multocida* were divided randomly into two groups of four individuals. In the infected group, the New Zealand rabbits were intraperitoneally injected with 1 mL of the prepared bacteria. In the control group, the New Zealand rabbits were intraperitoneally injected with 1 mL of PBS. The incidence and mortality of the New Zealand rabbits were observed, and lung samples were collected from the New Zealand rabbits for bacterial isolation and identification.

The cultured *P. multocida* was diluted using a 10-fold gradient with sterile saline at the final centrations of 10^9^, 10^8^, 10^7^, 10^6^, 10^5^, and 10^4^ CFU/mL. Fifty-six New Zealand rabbits were randomly divided into seven groups, with eight individuals in each group and intraperitoneally injected with different concentrations of *P. multocida* solution and 1 mL of PBS. Morbidity and mortality were observed for 15 consecutive days. The median lethal dose (LD 50) was calculated using the SPSS 19.0 software.

Twenty-five rabbits were randomly divided into the infected (*n* = 20) and control (*n* = 5) groups. Prior to the experimentation, the control group rabbits were euthanized by pentobarbital overdose (100 mg/kg, intravenous). The lung samples were collected from all freshly killed rabbits. The infected group was subcutaneously injected with 10^7^ CFU of *P. multocida* in 1 mL of PBS. After P1 and P3, every five rabbits with significant clinical symptoms of depression, anorexia, snuffles, serous nasal exudate, or dyspnea were euthanized, and the lung of each rabbit was dissected. All samples were collected aseptically, frozen in liquid nitrogen immediately, and transported to the lab, then stored at −80°C. The remaining alive rabbits, post-experiment, were euthanized by the same procedure.

### Total RNA Isolation and Qualification

Every three samples we used for the RNA-seq were taken at P0, P1, and P3 to examine the lung tissues at different stages of infection. Total RNA was extracted from the rabbits' lung tissues through a specialized Total RNA kit (Tiangen Biotech Co, Beijing, China) following the manufacturer's protocol. RNA concentration and purity were monitored using a NanoDrop 2000 Spectrophotometer (Thermo Fisher Scientific, Wilmington, DE, USA), and RNA integrity was checked using the Agilent 2100 Bioanalyzer (Thermo Fisher Scientific, MA, USA). The samples with RNA Integrity Number (RIN) ≥8 were subjected to further analysis.

### Library Construction and Sequencing

For the mRNA and lncRNA library, we firstly removed the ribosomal RNA (rRNA) using target-specific oligos, and we removed both the cytoplasmic and mitochondrial ribosomal RNA using RNase H reagents from the above total RNA samples. Secondly, the obtained RNA was fragmented into small pieces using divalent cations under elevated temperature to achieve a solid-phase reversible immobilization bead purification. Utilizing the cleaved RNA fragments, we synthesized the first-strand cDNA using random hexamer primers and reverse transcriptase, followed by the second-strand cDNA synthesis using DNA Polymerase I and RNase H. In this reaction, the RNA template was removed, and an alternative strand was synthesized. Moreover, dTTP was replaced by dUTP to generate the ds cDNA. These cDNA fragments had the addition of a single “A” base and subsequent ligation of the adapter. After the uracil-DNA glycosylase treatment, the incorporation of dUTP quenched the second strand during amplification. The products are enriched with PCR to create the final cDNA library.

Finally, the quality of the constructed libraries was assessed by checking the distribution of the fragment size using the Agilent 2100 Bioanalyzer, and their quantity was assessed using quantitative real-time PCR (qRT-PCR) (TaqMan Probe). The qualified libraries were sequenced on the BGISEQ-500 System (BGI-Shenzhen, China).

### Expression and Differential Expression Analysis

Raw reads were processed by removing the low-quality reads and adaptor contaminants, and other poly-N using the SOAPnuke (v1.5.2) (20), and the resulting clean reads were used for further study. The clean reads were aligned to the reference genome of *Oryctolagus cuniculus* 2.0 using both the HISAT2 (v2.0.4) (21) and Bowtie2 (v2.2.5) (22). The fragments per kilobase of transcript per million fragments mapped (FPKM) was used to quantify the expression levels of a gene or lncRNA calculated by the RSEM (v1.2.12) software (23). For the samples with biological replicates, DE analysis of lncRNAs and mRNAs in the three groups was determined by the DESeq2 (24) with a *Q* ≤ 0.05. The DESeq2 assumes a negative binomial distribution for gene counts, normalizes for read depths, and fits a generalized linear model.

### Gene Ontology and Kyoto Encyclopedia of Genes and Genomes Enrichment Analyses

Enrichment analysis of the DE genes was conducted using the Gene Ontology (GO, http://www.geneontology.org/) and Kyoto Encyclopedia of Genes and Genomes (KEGG, https://www.kegg.jp/) databases by Phyper (https://en.wikipedia.org/wiki/Hypergeometric_distribution) based on a hypergeometric test. The significant levels of terms and pathways were corrected using a Q value with a rigorous threshold (Q value ≤ 0.05) by Bonferroni (25). The GO terms or KEGG terms meeting this condition were considered as significantly enriched terms.

### qRT-PCR Validation

Total RNA was separated from the lung tissues using the Trizol Reagent (Takara Biotechnology, Dalian, China), and the cDNA was reversed from the RNA using a two-step qRT-PCR Kit (Takara Biotechnology, Dalian, China) following the manufacturer's instructions. The qRT-PCR reaction was conducted using the SYBR Green assay (Takara Biotechnology, Dalian, China) in the CFX96 Real-Time PCR Detection System (Bio-Rad) as previously described (11).

The specific quantitative primers of lncRNAs and mRNAs used in this study were either designed using the primer 6.0 software or cited from previously published literature (Table 1). All the primers were synthesized by Sangon Biotech (Sangon Biotech, Shanghai, China). Glyceraldehyde 3-phosphate dehydrogenase (GAPDH) was used as an endogenous control for the lncRNA and mRNA.

**Table 1:** The primers used in this experiment for qRT-PCR.

**Gene name**	**Sequence (5^**′**^-3^**′**^)**	**Production size (bp)**	**References**
TNF-α	F: CCAGATGGTCACCCTCAGAT	214	NM_001082263.1
	R: TTGACCGCTGAAGAGAACCT		
IL-1β	F: CAGGACCTGGACCTCTGCTGTC	103	NM_001082201.1
	R: GAGCCACAACGACTGACAAGACC		
CCL2	F: AGCACCAAGTGTCCCAAAGA	163	NM_001082294
	R: TGTGTTCTTGGGTTGTGGAA		
ISG15	F: GGACCTGAAGGTGAAGATGC	240	XM_017340429
	R: CTCAGCGGGTTGTCACACT		
IL6	F: GCACCTTCCAAGGCTGATAG	135	(26)
	R: CTCCTGAACTTGGCCTGAAG		
CXCL8	F: CTCTCTTGGCAACCTTCCTG	115	(26)
	R: GGATGGAAAGGTGTGGAGTG		
LOC108177705	F: AGACTACTCTGCGGTTGTGG	239	XR_516511
	R: AGTGACAGCTTCCATCCACA		
LOC108176536	F: CCTGACCGATGGTTTGTTCT	150	XR_001793202.1
	R: CGCATGCCTGTTTCATATTG		
LOC108177955	F: CCTGACCGATGGTTTGTTCT	150	XR_001794646
	R: CGCATGCCTGTTTCATATTG		
LOC108178511	F: GAACTCAGGGATGCTGCATG	209	XR_001795225.1
	R: ATGTCATATGCGGCCGTTTC		
LOC103351679	F: GCTGTGCTTGTACATCTGCC	221	XR_519410.2
	R: ATTGCAGGCTGAGGAGTTTG		
LOC108176706	F: TTTCCAGCACCATCCTTCTC	216	XR_001793425
	R: AGGCAGGAAGAACAGTTGGA		
GAPDH	F: TCACAATCTTCCAGGAGCGA	293	(27)
	R: CACAATGCCGAAGTGGTCGT		

### Statistical Analysis

The expression of mRNA and lncRNA with |log2FC| ≥ 0 and *Q* ≤ 0.05 were considered as significantly different on the RNA-seq analysis. The validation of each mRNA and lncRNA expression level was analyzed using the 2^−ΔΔCT^ method. qRT-PCR data were presented as means ± standard deviation (SD). All statistical analysis of the qRT-PCR data was performed using the SPSS version 19.0 software.

## Results

### *Pasteurella multocida* Identification

The series of experiments that we conducted showed that the presumptive bacteria was *P. multocida* of type A3. This strain can kill rabbits and re-isolate from infected rabbits. Moreover, rabbits infected with *P. multocida* can exhibit significant clinical symptoms. The LD 50 of this *P. multocida* strain is 2.3 × 10^7.8^ CFU/mL.

### Overview of lncRNA and mRNA Data

To clarify the effect of the rabbits' lncRNA and mRNA expression profiles during the *P. multocida* insults, we generated the RNA-seq data using the BGISEQ platform from nine lung samples of rabbits with or without the *P. multocida* infection, which obtained 114.94–119.94 million raw reads. After trimming the low-quality reads, adaptor sequences, and ambiguous nucleotides, 111.84–116.86 million clean reads passed a stringent quality filter and were yielded from each sample. More than 83% of the clean reads were successfully mapped to the rabbit reference genome, in which nearly 80% of the reads were mapped uniquely to the reference genome. In addition, nearly 80% of the clean reads of all nine samples had a quality score at the Q 30 level, which indicates an error chance of < 0.1% (Table 2) and suggests that the acquired were high-quality clean reads. We computed the pairwise Pearson' correlation coefficient (R) of theses samples at the whole-transcriptome level using a normalized gene expression to evaluate the individual variation. The results showed that there was a strong positive correlation across the samples, which ranged from 0.949 to 0.997.

**Table 2:** A summary of the alignment of sequencing reads to the oryctolagus cuniculus genome.

**Sample name**	**Total raw reads (M)**	**Total clean reads (M)**	**Clean reads Q30 (%)**	**Total mapping genome ration (%)**	**Uniquely mapping genome ratio (%)**
P0-1	117.44	114.14	89.07	83.73	80.35
P0-2	119.94	116.85	89.82	84.29	81.31
P0-3	119.94	116.52	88.81	83.19	79.88
P1-1	117.44	114.20	89.06	83.54	80.05
P1-2	119.94	116.63	89.04	83.65	79.55
P1-3	119.64	116.30	88.81	83.72	79.90
P3-1	119.94	116.86	89.23	84.11	81.42
P3-2	114.94	111.84	89.19	83.48	79.79
P3-3	119.94	116.76	89.09	83.46	79.80

Finally, a total of 2,334 lncRNAs, of which, 2,081 were known and 253 were novel (Supplementary Data Sheet 1), and 17,758 mRNAs, of which, 14,499 were known and 3,259 were novel (Supplementary Data Sheet 2), were detected. Three comparison groups were set based on the different times during the *P. multocida* infection, including P0-vs. (vs.)-P1, P0-vs.-P3, and P3-vs.-P1, and the following analyses were based on these transcripts among the three comparison groups. The RNA-seq data have been deposited in the NCBI BioProject database under accession number PRJNA648635.

### Differential Expression Analysis of lncRNAs and mRNAs

To identify the high-confidence of DE lncRNAs and mRNAs, the DESeq2(v1.4.5) software with a strict criterion of |log2FC| ≥ 0 and *Q* ≤ 0.05 was applied to screen by FPKM. Firstly, 336 significantly DE lncRNAs (Supplementary Data Sheet 3) were found for the pairwise comparisons between the P0, P1, and P3 of the samples collected from rabbit lungs, with 35 lncRNAs (19 upregulated and 16 downregulated) in the P0-vs.-P1 group, 268 lncRNAs (141 upregulated and 127 downregulated) in the P0-vs.-P3 group, and 243 lncRNAs (114 upregulated and 129 downregulated) in the P3-vs.-P1 group.

Secondly, 7,014 significantly DE mRNAs (Supplementary Data Sheet 4) were identified, with 874 mRNAs (418 upregulated and 456 downregulated) in the P0-vs.-P1 group, 5,807 mRNAs (2,975 upregulated and 2,832 downregulated) in the P0-vs.-P3 group, and 5,295 mRNAs (2,849 upregulated and 2,446 downregulated) in the P3-vs.-P1 group. However, this finding was similar to those of other reports, with the average expression levels of mRNA being much higher than that of the identified lncRNAs (28, 29).

**Figure 1:**
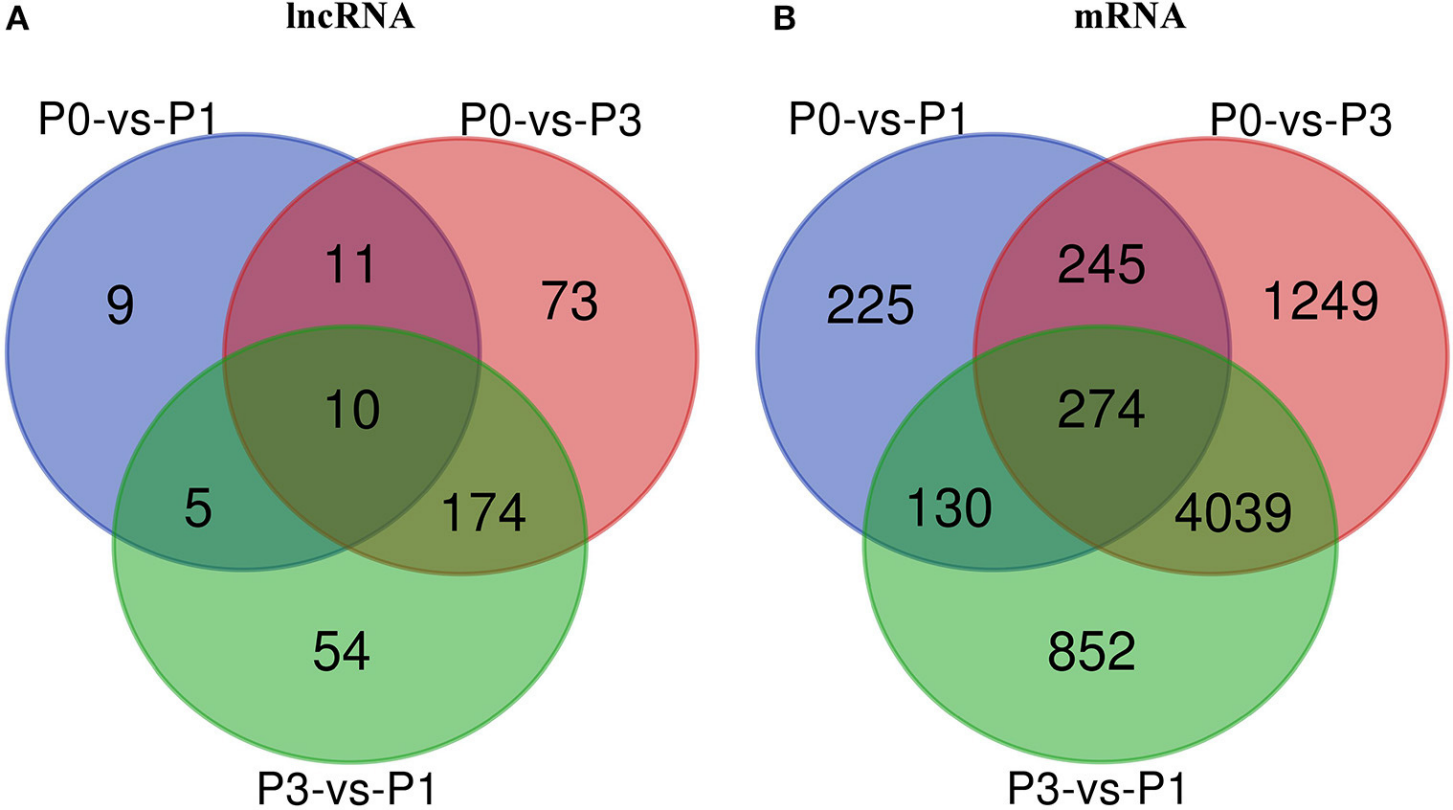
Venn diagram showing the common DE lncRNAs **(A)** and mRNAs **(B)** in the three comparison groups: P0-vs.-P1, P0-vs.-P3, and P3-vs.-P1.

**Figure 2:**
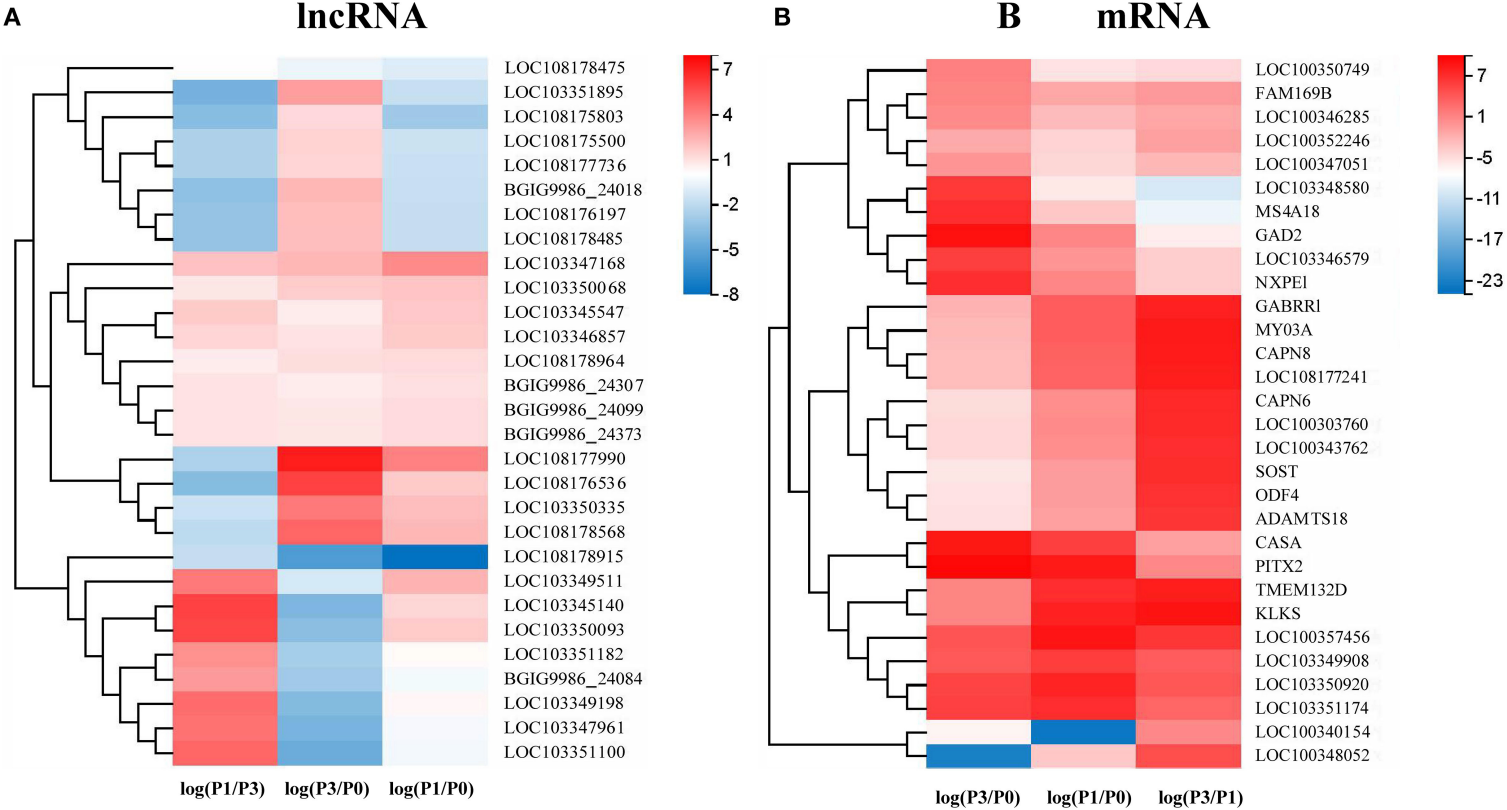
A hierarchical heat map showing the relative expression value of the top 10 specific intergroup DE lncRNAs **(A)** and mRNAs **(B)** in the three comparison groups: P0-vs.-P1, P0-vs.-P3, and P3-vs.-P1 (the number of DE lncRNA in the P0-vs.-P1 are nine).

A Venn diagram was created to depict the overlaps among the DE lncRNAs and mRNAs from the pairwise comparisons. In total, 9, 73, and 54 specific intergroup DE lncRNAs were discovered in the P0-vs.-P1, P0-vs.-P3, and P3-vs.-P1 groups, respectively (Figure 1A). A hierarchical heat map showed that the most upregulated specific intergroup lncRNAs were LOC108177990 (6.16), LOC103347168 (2.90), and LOC103345140 (5.02), and the most downregulated specific intergroup lncRNAs were LOC108178915 (−5.71), LOC108178475 (−1.47), and LOC103351895 (−4.54) in the three comparison groups, respectively (Figure 2A). In addition, 10 dysregulated lncRNAs were shared among the three comparison groups (Figure 1A). Moreover, a set of 225, 1,249, and 852 dysregulated mRNAs were the specific intergroup expressed in the P0-vs.-P1, P0-vs.-P3, and P3-vs.-P1 groups, respectively (Figure 1B). The highest upregulated specific intergroup mRNAs were PITX2 (6.60), LOC100357456 (5.78), and KLK5 (5.845), and the lowest downregulated specific intergroup mRNAs were LOC100348052 (−21.37), LOC100340154 (−22.28), and LOC103348580 (−10.33) in the three comparison groups, respectively (Figure 2B). Furthermore, 274 dysregulated mRNAs were identified as the transcripts shared among the three comparison groups (Figure 1B). In general, the set of lncRNAs and mRNAs suggested the existence of global coordination for the regulatory responses in the rabbit lung immune response to *P. multocida* type A infection.

### Delineation of Go Annotations and KEGG Pathways Analysis

To further predict the functions of DE mRNAs during the *P. multocida* type A infection at different times, we performed the systematic GO and KEGG analysis. The GO functional enrichment analysis for the dysregulated DE mRNAs of rabbit lung after the infection by *P. multocida* type A covered three distinct aspects: biological process (BP), cellular component (CC), and molecular function (MF). The results revealed that a total of 37, 55, and 59 highly enriched GO terms were derived from P0-vs.-P1 (Supplementary Data Sheet 5), P0-vs.-P3 (Supplementary Data Sheet 6), and P3-vs.-P1 (Supplementary Data Sheet 7), respectively (*Q* < 0.05). Furthermore, the highly enriched GO terms in the three comparison groups covered a diverse range of functional categories, particularly a variety of immune-related functions: inflammatory response, innate immune response, immune response, response to virus, and chemokine activity.

Through the KEGG analysis, a total of 7, 61, and 49 significantly enriched KEGG pathways were derived from P0-vs.-P1, P0-vs.-P3, and P3-vs.-P1 (Supplementary Data Sheet 8), respectively (*Q* < 0.05). We also noticed that the most significantly enriched pathways of the DE mRNAs included the pathways in cancer and ribosome biogenesis in eukaryotes, which were the same in the P0-vs.-P3 and P3-vs.-P1 groups (Supplementary Data Sheet 8). Furthermore, it was notable that, compared with the P0-vs.-P1 group, the P0-vs.-P3 group showed a higher number of enriched immune-related pathways. In addition, the DE mRNAs in the P0-vs.-P3 group were significantly enriched in the immune system and signal transduction signaling pathway (Table 3). Overall, the vital roles of immune-related mRNAs that dysregulated during the invasion in the P0-vs.-P3 might be relevant to the induction of those genes in the regulation process of the rabbit immune response to *P. multocida* type A infection.

**Table 3:** Association between the immune and signal transduction pathway in the lung transcriptome of rabbits infected with *P. multocida* type A at 3 dpi.

**Pathway**	**Ko ID**	***Q*-value**	**Number of DEGs**
TNF signaling pathway	04668	1.32E-08	63
NOD-like receptor signaling pathway	04621	4.04E-08	88
NF-kappa B signaling pathway	04064	6.24E-08	53
Toll-like receptor signaling pathway	04620	2.10E-05	51
IL-17 signaling pathway	04657	3.03E-05	50
MAPK signaling pathway	04010	7.74E-04	123
Influenza A	05164	0.001138692	79
C-type lectin receptor signaling pathway	04625	0.001933125	48
Chemokine signaling pathway	04062	0.004889786	76
Cytokine-cytokine receptor interaction	04060	0.009346174	111
Wnt signaling pathway	04310	0.01560301	64
RIG-I-like receptor signaling pathway	04622	0.02460512	34
PI3K-Akt signaling pathway	04151	0.02621846	126

### The Signaling Pathway Involved in the Immune System During *P. multocida* Infection

**Figure 3:**
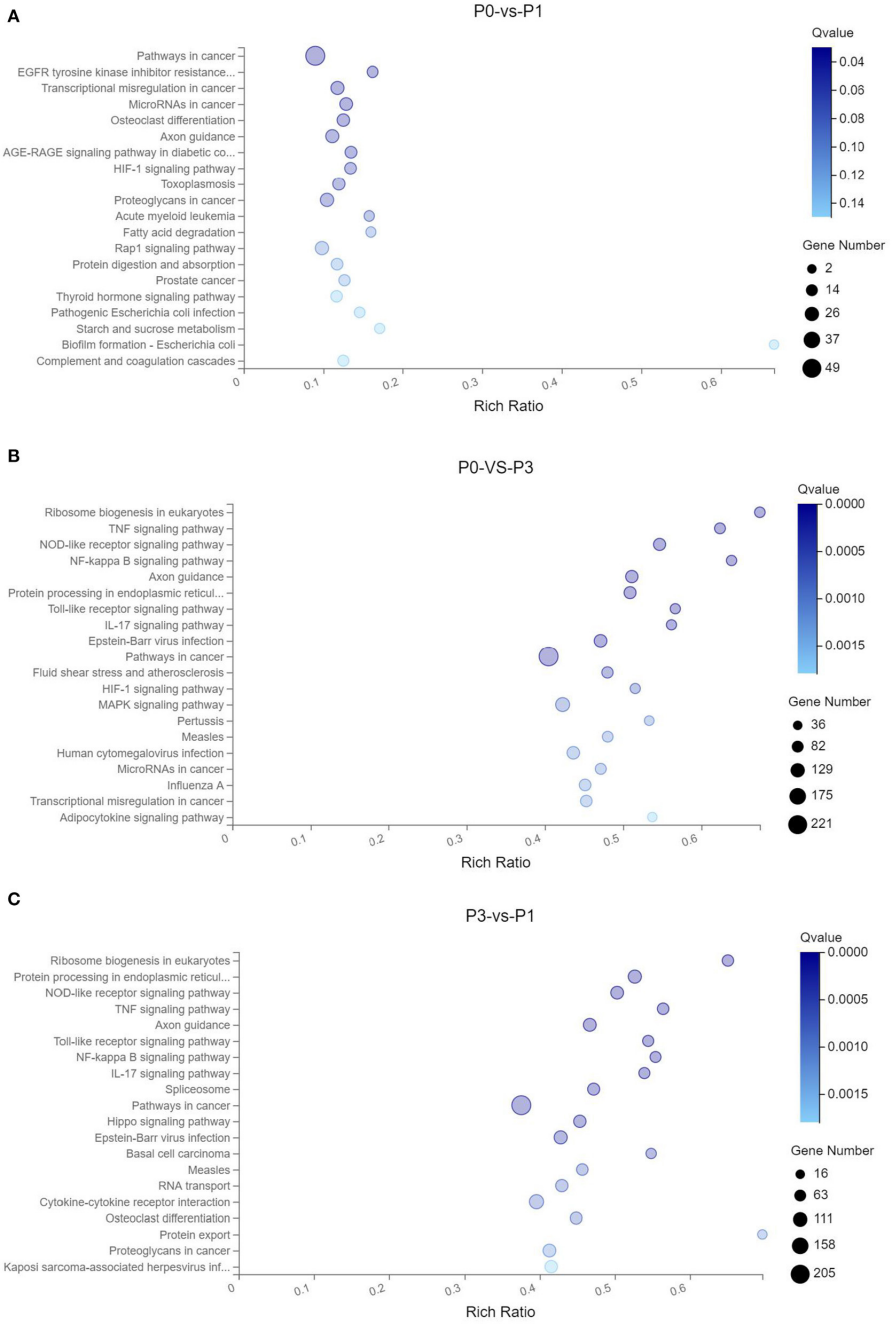
KEGG pathway enrichment analysis of the DE mRNAs with the 20 highest rich ratios in the three comparison groups **(A)** P0-vs.-P1, **(B)** P0-vs.-P3, and **(C)** P3-vs.-P1. The abscissa showed the rich ratio and the ordinates showed the detailed terms. The size of dots indicates the number of enriched genes, and the color of dots indicates the degree of enrichment. Higher rich ratio correlate with lower Q-values, indicating that the enrichment of DE genes in a given pathway is significant.

**Figure 4:**
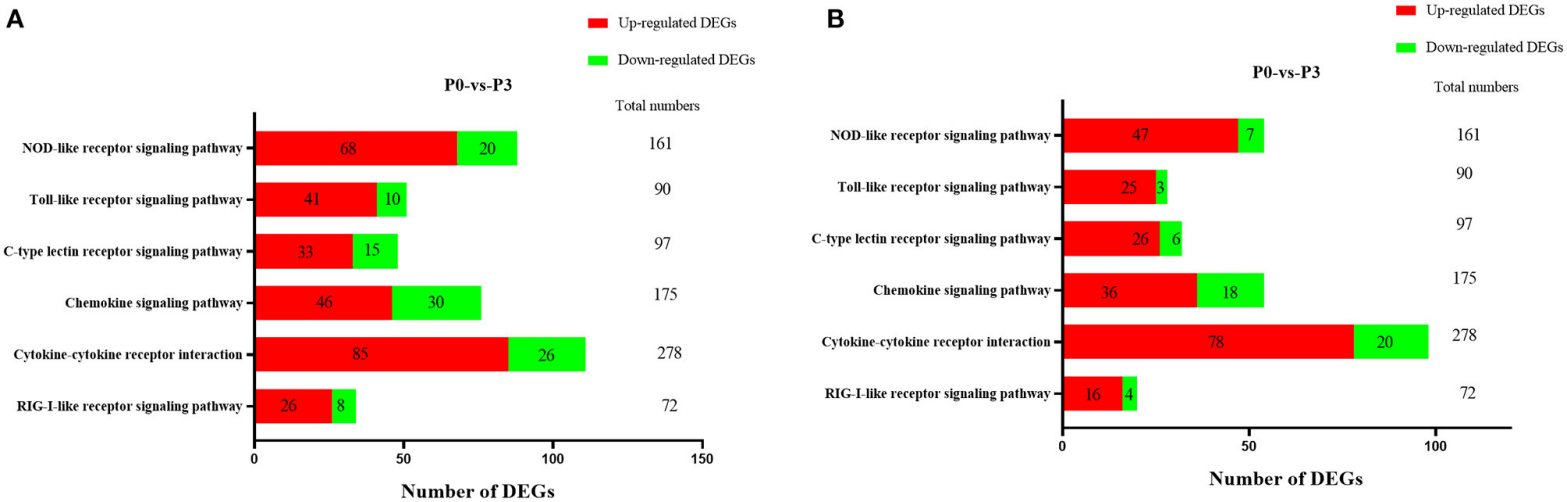
The crucial immune system pathways enriched during *P. multocida* infection at 3 dpi. **(A)** DEGs with fold changes ≥1, **(B)** DEGs with fold changes ≥2.

To clarify the crucial pathways involved in *P. multocida* infection, immune-related signaling pathways were further screened among the three groups (Figures 3A–C), especially in the P0-vs.-P3 group (Figure 3B). These were considerably enriched in the immune-related pathways, including the NOD-like receptor signaling pathway, TLR signaling pathway, C-type lectin receptor signaling pathway, chemokine signaling pathway, cytokine-cytokine receptor interaction, and RIG-I-like receptor signaling pathway (Table 3 and Supplementary Data Sheet 9). In addition, most immune-related differentially expressed genes (DEGs) were found to be upregulated in the P0-vs.-P3 group, especially DEGs with fold changes of ≥2 (Figure 4B).

### Expression of Key DE mRNAs in Immune-Related Pathways During *P. multocida* Infection

**Figure 5:**
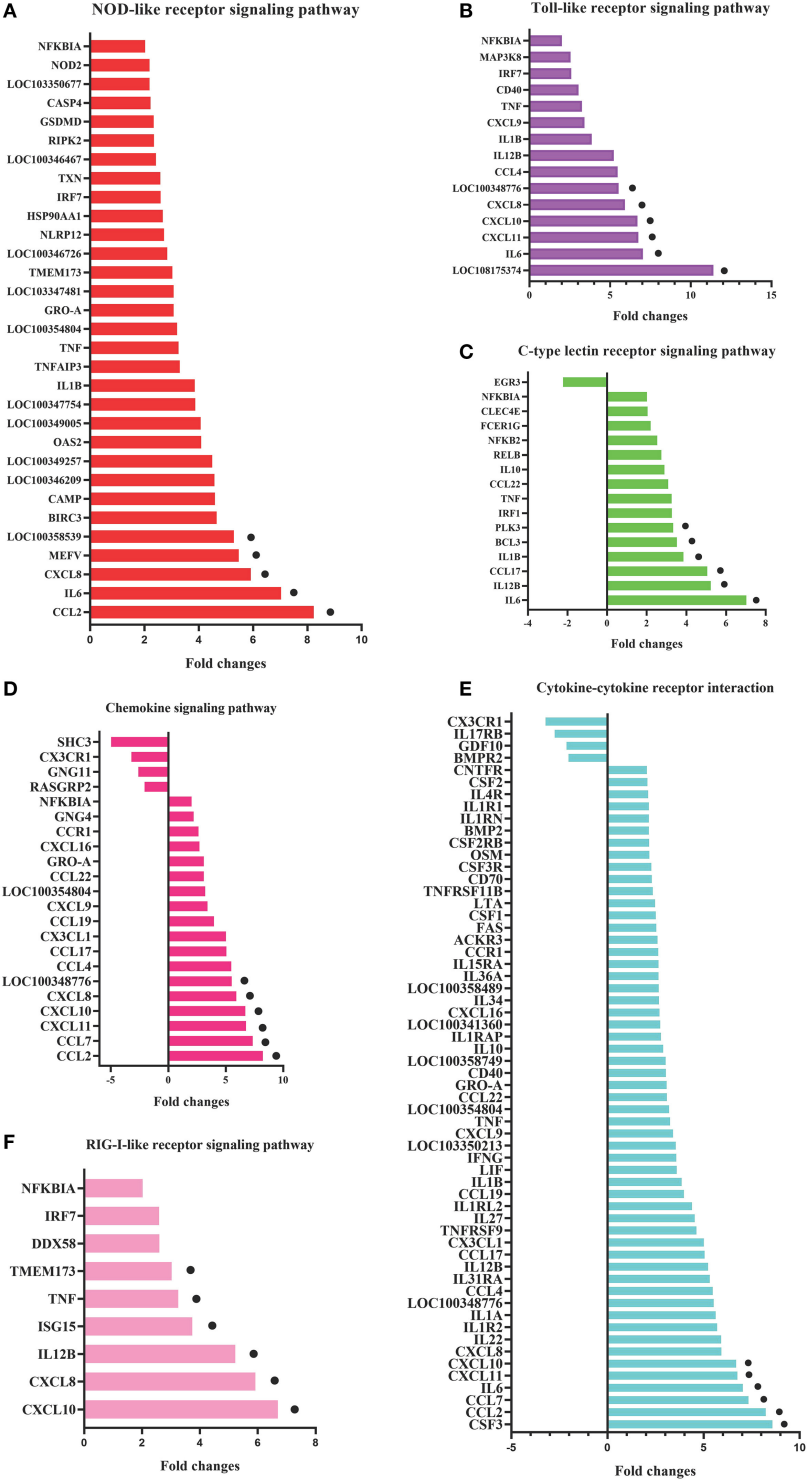
DEGs in the six most enriched immune-related signaling pathways in the P0-vs.-P3 group with the fold changes ≥4. DEGs in the NOD-like receptor signaling pathway **(A)**, Toll-like receptor signaling pathway **(B)**, C-type lectin receptor signaling pathway **(C)**, chemokine signaling pathway **(D)**, cytokine-cytokine receptor interaction **(E)**, and RIG-I-like receptor signaling pathway **(F)**. The six DEGs with the most substantial fold changes in expression in each pathway marked by black dots.

To identify the crucial genes in the immune-related pathways, the expression of genes with larger fold changes upon *P. multocida* infection were screened. The top six DEGs were different for the different signaling pathways: for the NOD-like receptor signaling pathway, they were CCL2, IL6, XCCL8, MEFV, LOC100358539, and BIRC3; for the TLR signaling pathway, they were LOC108175374, IL6, CXCL11, CXCL10, CXCL8, and LOC100348776; for the C-type lectin receptor signaling pathway, they were IL6, IL12B, CCL17, IL1B, BCL3, and PLK3; for the chemokine signaling pathway were CCL2, CCL7, CXCL11, CXCL10, CXCL8, and LOC100348776; for the cytokine-cytokine receptor interaction, they were CSF3, CCL2, CCL7, IL6, CXCL11, and CXCL10; and for the RIG-I-like receptor signaling pathway, they were CXCL10, CXCL8, IL12B, ISG15, TNF, and TMEM173 (Figure 5).

### Verification by qRT-PCR

**Figure 6:**
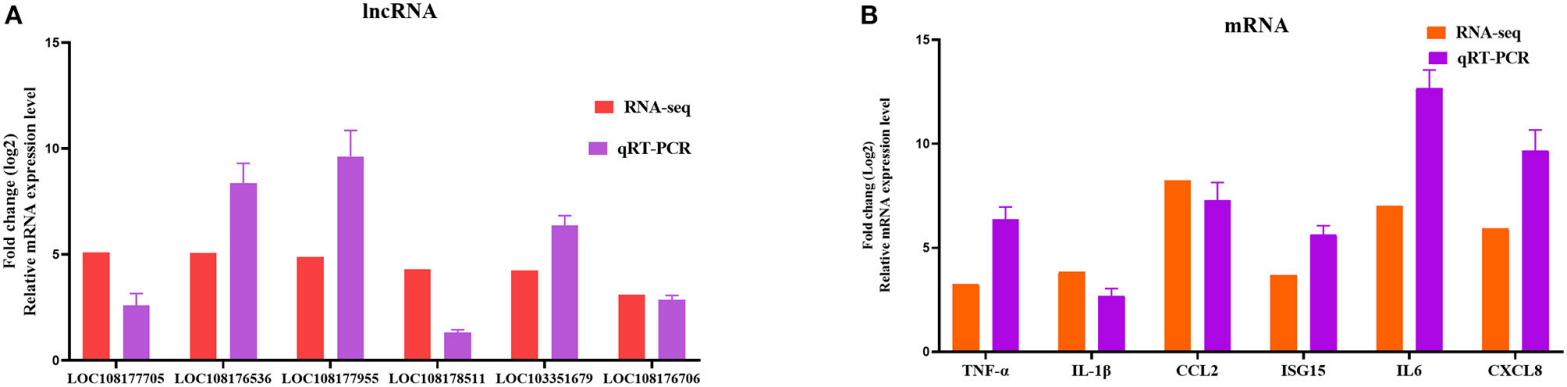
Validation of the RNA-seq results by qRT-PCR. Six DE lncRNAs **(A)** and mRNAs **(B)** that participated in immune-related events were picked for qRT-PCR analysis, and expression was quantized using the 2^−ΔΔCT^ method. Data for five independent trials were exhibited as mean ± SD.

To further assess the accuracy and reliability of the RNA-seq results, we conducted additional qRT-PCR experiments with six DE lncRNAs and six DE mRNAs selected based on the crucial immune-related BP in the P0-vs.-P3 comparison group. As shown in Figure 6, the data exhibited that the RNA-seq was well correlated with our qRT-PCR results.

## Discussion

Rabbits are important target animals for *P. multocida* infection, which leads to a decrease in the production efficiency and ultimately brings a considerable economic loss (4). In China, A-type *P. multocida* is the most prevalent in rabbits, and the lungs are the primary target organs (30, 31). However, the underlying molecular pathogenesis mechanisms of pathogen-host interactions in response to this microbe attack are still elusive. Owing to the development of a convenient high-throughput sequencing technology and the progress of reliable bioinformatics, regardless of mRNAs, increasing ncRNAs, such as miRNAs and lncRNAs, especially lots of novel ncRNAs were discovered at the transcriptional level, which provides a new perspective for clarifying the molecular pathogenesis mechanisms and unique BPs of host-pathogen interactions (32). A wide range of pathogens can induce and regulate the DE mRNAs and ncRNAs upon infection (12, 33, 34). However, so far as is known, no reports have examined the lncRNA and mRNA expression profiles of the interaction between *P. multocida* and rabbit lungs. In this research, we firstly identified that the presumptive bacteria were *P. multocida* of type A3, with the aim to depict the genetic architecture of rabbit lung transcriptomes to further facilitate in illuminating the molecular mechanism of *P. multocida* attacks. Hence, we conducted the lncRNAs and mRNAs examinations on healthy rabbit lungs (0 dpi), and on rabbit lungs at 1 dpi and 3 dpi. For the first research of lncRNAs in *P. multocida* attacks of rabbits, we found that certain lncRNAs and mRNAs were significantly dysregulated.

LncRNAs are defined as transcripts longer than 200 nucleotides, but they lack a protein-coding function (13). The characteristics of lncRNAs are similar to mRNA after being post-transcriptionally capped and polyadenylated (15). Unlike mRNA, which has been extensively investigated in *P. multocida* (35, 36), the function of most lncRNAs and their potential roles in *P. multocida* infection are poorly explained. Based on the sequences, structures, and subcellular localization, lncRNAs display versatile functions of regulator gene expression splicing, nucleic acid degradation that interacts with DNA, RNA, and proteins (37). Moreover, lncRNAs have existed in manifold immune cells, such as monocytes, macrophages, dendritic cells, neutrophils, T cells, and B cells, which are induced to modulate the innate and adaptive immune responses in the pathogen-host interaction acting as scaffolds, decoys, guides, or signals (12, 38). Current research has indicated that microbe infection can induce lncRNAs to promote or inhibit microbe response. For example, lncRNA MEG3-4 serves as a miRNA decoy that regulates IL-1β abundance to initiate and then limit inflammation to prevent sepsis during lung infection (39). In mammals, lncRNAs are the most abundant class of ncRNA, but the least understood. Using a paired-end sequencing, a total of 2,334 lncRNAs were detected, of which, 235 were novel lncRNAs (Supplementary Data Sheet 1), as expressed in the rabbit lung before and after the *P. multocida* infection. Among them, the expression of 336 lncRNAs changed more than 1-fold, and 250 lncRNAs changed more than 2-fold (Supplementary Data Sheet 3). A notable feature of lncRNA changes in expression was that the most striking dysregulation occurred at the P3, with nearly 80% of the DE lncRNAs exhibited an increased expression (Figure 1A), suggesting that the *P. multocida* genes are responsible for the regulation. We also observed that certain lncRNAs significantly increased during P3, which included LOC108177705, LOC108177990, LOC108175524, LOC103348070, LOC108176536, and BGIG9986_24083 (Figure 2A). Mounting evidence from the available data suggests that the expression of the dysregulated lncRNAs can regulate and be highly related to the expression of neighboring mRNAs (18). However, the classification and biological functions of the rabbit lncRNAs are unclear. Therefore, further study is needed to explore the fundamental characteristics and biological function of lncRNAs in rabbit *P. multocida* initiation and progression.

In the present study, rabbits infected with *P. multocida* exhibited evident clinical signals which are consistent with previous literature (1, 4). The current understanding of the interaction between *P. multocida* and its host implies that the microbe builds an intricate interplay in host organs and makes use of its available niche to proliferate rapidly and lead to diseases in beneficial conditions (40). Moreover, we observed that the expression of 7,014 mRNAs changed more than 1-fold and 3,470 mRNAs changed more than 2-fold (Supplementary Data Sheet 4). In addition, similar to the lncRNAs, the highest number of DEGs were observed at P3, especially those that were immune-related. The increase in the number of DEGs during *P. multocida* infection suggests that in rabbits, a growing number of genes are activated in response to bacterial replication. Therefore, the following discussion focuses primarily on the immune-related gene in response to *P. multocida*, which included PRRs, downstream pathways of PRRs, and effector molecules, such as cytokines and chemokines.

Innate PPRs, which mediate the initiation immune signaling, play pivotal roles in defending against extrinsic pathogens infection via recognizing the pathogen-associated molecular patterns (41). At P3, the KEGG signaling pathway annotations showed that various DEGs were involved in the TLR signaling pathway, NOD-like receptor signaling pathway, C-type lectin receptor signaling pathway, and RIG-I-like receptor signaling pathway. The remarkable feature of the DE mRNAs at P3 was that the number of upregulated genes were significantly higher than the number of downregulated genes (Figure 4). In the TLR signaling pathway, the expressions of TLR2, TLR3, TLR4, and TLR5 were all significantly upregulated at P3. Furthermore, NOD1, NOD2, and NLRP3 increased considerably at the transcriptional level at P3. Activation of TLRs through the MyD88-dependent and independent pathways induced a series of kinase phosphorylations and gave rise to NF-κB activation (42). The NLRs family, which is comprised of NOD1, NOD2, and NLRP3 (NOD-like receptor family, pyrin domain-containing, NLRP), are similar to those of the TLRs. Both NOD1 and NOD2 activate the NF-κB and MAPK signal pathway against bacteria (43). In addition, the activation of NLRP3 inflammasome triggers the release of mature IL-18 and IL-1β (44). Similar to the role of TLRs, *Escherichia coli, Chlamydophila pneumoniae, Campylobacter jejuni*, and *Listeria monocytogenes* are recognized by NOD1, while *Streptococcus pneumonia* and *Mycobacterium tuberculosis* are sensed by NOD2 (45). Other data also demonstrated that NLRP3 inflammasome exerts a crucial function in caspase-1 activation and IL-1β production during the *P. multocida* invasion of macrophages (46).

Following recognition by PRRs, the down effector pathway mediated cascades involving NF-κB, and the MAPK signaling pathways were enriched by the KEGG analysis. A total of 123 (fold > 1) DEGs were identified to have participated in the MAPK signal pathway and 53 (fold > 1) DEGs in the NF-κB signaling pathway (Supplementary Data Sheet 9). Diverse microbes including *E. faecalis, S. aureus*, and *M. tuberculosis*, have been reported to activate the NF-κB and MAPK signaling pathways to regulate the secretion of pro-inflammatory cytokines, which play essential functions in fighting bacterial attacks (47–49). Hence, NF-κB and MAPK signaling may also play vital roles during *P. multocida* attacks. Usually, the threshold for the reliable quantitation of the DE mRNAs and non-coding RNAs was set at |log2FC| ≥ 1 and *Q* ≤ 0.05. In our statistical analysis, we set the reliable threshold as |log2FC| ≥ 0 and *Q* ≤ 0.05. Due to the lowering of the threshold, the results will significantly increase the type I error. Thus, the false positive rate will be increased throughout the differential expression results (50–52). Therefore, in the following analysis, we mainly focused on the DE genes that had fold changes ≥2.

Cytokines are vital immunomodulators that are secreted in response to injury or inflammation, which restrict or find balance between the pro-inflammatory and anti-inflammatory cytokines. Cytokines also play essential roles in host defense against microbial pathogens (53). Based on the KEGG enrichment analysis, many DEGs participated in cytokine mediating signal in response to the *P. multocida* insults at P3. We found that 111 DEGs were included in the cytokine-cytokine receptor interaction and 85 DEGs were upregulated. Furthermore, the top six DEGs, including CSF3, CCL2, CCL7, IL6, CXCL11, and CXCL10, were all upregulated after infection at P3 (Supplementary Data Sheet 9). In this research, these types of upregulated cytokines bear some similarity to mouse lungs that have been infected by *P. multocida* (35, 54). Therefore, we assumed that changes in the immunological characteristics of mice in response to *P. multocida* might be similar to rabbits in some aspects. However, further research is needed to clarify the similarity and difference between the rabbit and mice pathogenesis mechanism during a *P. multocida* infection.

Except for the cytokine-cytokine receptor interaction, the chemokine signaling pathway is as well-crucial in response to *P. multocida* attacks based on the KEGG annotation. Chemokines are a large family of small cytokines that are characterized by the presence of N-terminal cysteine residues, which are primarily divided into C-X-C and C-C subfamilies. The C-X-C subfamily is focused on inducing chemotaxis for neutrophils. However, the C-C subfamily is concentrated on inducing chemotaxis for monocytes and specific lymphocytes subsets. C-X-C chemokines play a crucial function upon diverse bacterial (*P. aeruginosa, M. tuberculosis, L. monocytogenes*, and *Helicobacter pylori*) attacks through influencing the neutrophils (55–58). In our data, we identified eight CC DEGs (fold > 2), including CCL2, CCL7, CCL4, CCL17, CCL22, CCL14, and CCL21. In addition to CCL21, all the other CC increased in the transcriptional level. Moreover, 5 C-X-C DEGs (fold > 2) are up-regulated, including CXCL11, CXCL10, CXCL8, CXCL9, and CXCL16. Only CXCL12 was downregulated (Supplementary Data Sheet 9). Overall, our data strongly indicate that there might be a linkage between the chemokine gene expression and *P. multocida* attacks. However, the precise molecular regulation mechanism of chemokines in fighting against the *P. multocida* attacks needs further illumination.

## Conclusions

Here, we report the first comprehensive description of the effect of *P. multocida* attacks on rabbit lncRNAs and mRNAs gene expression. The data showed that challenge by how *P. multocida* elicited a dramatic change in the lncRNAs and mRNAs gene expression in rabbit lungs. A further detailed investigation of the functions of lncRNAs in immune evasion and *P. multocida* pathogenesis could provide valuable clues to the prevention and control of *P. multocida* infections. Moreover, the immune-related genes that changed are mainly related to the PRRs, down effector molecules, cytokines, and chemokines, suggesting the intricate immune response in the rabbit lung during *P. multocida* attacks. This research provides a novel, comprehensive understanding of lncRNAs and mRNA interacting with *P. multocida*.

## Data Availability Statement

The RNA-seq data have been deposited in the NCBI BioProject database under accession number PRJNA648635.

## Ethics Statement

All animal studies in this article were performed according to the guidelines of the Institutional Animal Care and Use Committee of Shandong Agricultural University and the Guidelines for Experimental Animals of the Ministry of Science and Technology (Beijing, China).

## Author Contributions

JH, XF, BH, and QZ designed this study. JH and WL took the samples, conducted the experiments, and write the manuscript. XF modified the manuscript. All authors contributed to the article and approved the submitted version.

## Conflict of Interest

The authors declare that the research was conducted in the absence of any commercial or financial relationships that could be construed as a potential conflict of interest.

## Supplementary Material

Supplementary Data Sheet 1 Detailed information of the detected lncRNAs.

Supplementary Data Sheet 2 Detailed information of the detected mRNAs.

Supplementary Data Sheet 3 Detailed information of the DE lncRNAs.

Supplementary Data Sheet 4 Detailed information of the DE mRNAs.

Supplementary Data Sheet 5 Detailed information of GO enrichment of DE mRNAs in the P0-vs-P1 group.

Supplementary Data Sheet 6 Detailed information of GO enrichment of DE mRNAs in the P0-vs-P3 group.

Supplementary Data Sheet 7 Detailed information of GO enrichment of DE mRNAs in the P3-vs-P1 group.

Supplementary Data Sheet 8 Detailed information of KEGG enrichment of DE mRNAs.

Supplementary Data Sheet 9 Detailed information of the major immune-related signal pathway.
